# Hepatic-Predominant Immunoglobulin G Lambda Light Chain Amyloidosis Associated With Multiple Myeloma: A Report of an Exceptionally Rare Case

**DOI:** 10.7759/cureus.101793

**Published:** 2026-01-18

**Authors:** Guilherme Jesus, Inês Soares, Ana Sofia Silva, Mariana Baptista, Ana Gonçalves Ribeiro

**Affiliations:** 1 Internal Medicine, Unidade Local de Saúde Gaia e Espinho, Vila Nova de Gaia, PRT; 2 Anatomic Pathology, Unidade Local de Saúde Gaia e Espinho, Vila Nova de Gaia, PRT

**Keywords:** amyloid light chain amyloidosis, daratumumab, hepatic amyloidosis, multiple myeloma, plasma cell dyscrasia

## Abstract

Amyloid light chain (AL) amyloidosis represents a rare plasma cell dyscrasia characterized by the deposition of misfolded immunoglobulin light chains in various organs. While hepatic involvement is often detected histologically in systemic AL amyloidosis, symptomatic hepatic presentation as the dominant initial manifestation remains uncommon. We present the case of a 65-year-old woman who developed progressive constitutional symptoms, cholestatic jaundice, and hepatomegaly as the initial manifestations of immunoglobulin G (IgG) lambda light chain amyloidosis associated with multiple myeloma. The patient presented with a six-month history of weight loss exceeding 20 kilograms, daily vomiting, night sweats, and progressive jaundice. Laboratory investigations revealed marked cholestasis, with an alkaline phosphatase level of 1252 U/L, a gamma-glutamyl transferase level of 1426 U/L, hyperbilirubinemia, and coagulopathy. Serum immunofixation electrophoresis demonstrated an IgG lambda monoclonal protein (M-protein). Abdominal fat pad biopsy confirmed lambda light chain amyloid deposition, while bone marrow examination revealed 30% plasma cell infiltration consistent with multiple myeloma. Echocardiography demonstrated findings suggestive of cardiac involvement with reduced global longitudinal strain and apical sparing pattern. Notably, renal function remained preserved without proteinuria, and skeletal imaging showed no lytic lesions. The patient was treated with daratumumab in combination with cyclophosphamide, bortezomib, and dexamethasone (Dara-VCD), demonstrating a very good partial response (VGPR) with 75% reduction of M-protein (from 1.2 g/L to 0.3 g/L) after four treatment cycles. This case exemplifies a clinically significant presentation combining IgG lambda-type multiple myeloma, systemic AL amyloidosis, and dominant hepatic involvement as the initial clinical manifestation. The combination of these features is rarely reported in the medical literature, making this case instructive for recognizing atypical presentations that may lead to diagnostic delays. Early recognition and prompt initiation of plasma cell-directed therapy are essential for improving outcomes in this challenging condition.

## Introduction

Amyloid light chain (AL) amyloidosis constitutes the most common form of systemic amyloidosis in developed countries, with an estimated annual incidence of 9-14 cases per million population [1]. This disorder arises from clonal plasma cells producing misfolded immunoglobulin light chains that aggregate and deposit as insoluble amyloid fibrils in various tissues, leading to progressive organ dysfunction [2]. Approximately 10-15% of patients with multiple myeloma develop clinically significant AL amyloidosis, although the true prevalence may be higher when including subclinical cases [3]. The coexistence of multiple myeloma and AL amyloidosis portends a particularly grave prognosis, with median survival ranging from eight to nine months in historical cohorts, though outcomes have improved with modern therapies [4].

The clinical presentation of AL amyloidosis varies considerably depending on the pattern and extent of organ involvement. The most commonly affected organs include the kidneys (70% of cases), heart (50-60% of cases), liver (15-30% symptomatic involvement), and peripheral nervous system [5]. Hepatic amyloidosis, while frequently detected histologically at autopsy (occurring in 56-95% of cases), manifests as clinically dominant disease in only approximately 5% of patients, and isolated hepatic presentation occurs in merely 1% of cases [6]. The typical clinical manifestations of hepatic amyloidosis include hepatomegaly, alkaline phosphatase elevation, and, less commonly, jaundice or coagulopathy [7]. Multiple myeloma associated with immunoglobulin G (IgG) lambda light chains represents a less common variant compared to IgG kappa light chains. In the general population of multiple myeloma patients, IgG kappa occurs approximately twice as frequently as IgG lambda [8]. When AL amyloidosis develops in the context of IgG-producing myeloma, lambda light chains are involved in approximately 70-80% of cases, maintaining the general predominance of lambda over kappa seen in AL amyloidosis overall [8]. However, the specific combination of multiple myeloma with IgG lambda monoclonal protein (M-protein), systemic AL amyloidosis, and primary hepatic presentation is rarely reported, with the constellation of IgG lambda-type disease with hepatic-predominant involvement and sparing of renal involvement being an uncommon clinical scenario [9].

We present the case of a 65-year-old woman who developed severe constitutional symptoms with dominant hepatic manifestations as the initial presentation of IgG lambda multiple myeloma complicated by systemic AL amyloidosis. This case highlights the diagnostic challenges inherent in recognizing atypical presentations of plasma cell disorders and emphasizes the importance of maintaining clinical suspicion for amyloidosis in patients presenting with unexplained multi-organ dysfunction.

## Case presentation

A 65-year-old previously independent woman presented to the emergency department with progressive constitutional symptoms and laboratory abnormalities detected during outpatient evaluation. She had been employed as a domestic worker prior to symptom onset. Her medical history included well-controlled hypertension, type 2 diabetes mellitus with glycated hemoglobin of 5.4%, dyslipidemia, beta-thalassemia minor (baseline hemoglobin approximately 10.5-11.5 g/dL), depression, anxiety, and urticaria. Surgical history was notable for total hysterectomy and cholecystectomy performed in previous years. Family history revealed two brothers with prostate cancer, one sister who died from uterine cancer at approximately 62 years of age, and a maternal grandmother with intestinal cancer. Tobacco or alcohol use was denied.

The patient had been followed in primary care and in the hospital internal medicine outpatient clinic for approximately six months with progressive symptoms including drenching night sweats, colicky right upper quadrant abdominal pain, daily post-prandial vomiting (particularly after breakfast), profound fatigue, and dramatic weight loss exceeding 20 kilograms. Approximately two weeks prior to emergency department presentation, the patient developed clinically apparent jaundice of the skin and mucous membranes. She denied dysuria, hematuria, or constitutional fever, though she reported subjective episodes of diaphoresis. Upper and lower gastrointestinal endoscopy had been performed approximately one year prior to presentation with unremarkable findings.

On admission to the hospital, the patient appeared chronically ill but was oriented and cooperative. Vital signs revealed blood pressure of 126/89 mmHg, heart rate of 89 beats per minute, temperature of 36.9 degrees Celsius, and oxygen saturation of 98% on room air. Physical examination was remarkable for marked scleral and cutaneous icterus. Cardiovascular examination demonstrated regular rhythm without murmurs. Respiratory examination revealed clear lung fields bilaterally. Abdominal examination was significant for a globular, distended abdomen with palpable hepatomegaly and discomfort on deep palpation of the right upper quadrant without peritoneal signs. Lower extremities showed no peripheral edema. Neurological examination was unremarkable with normal cranial nerve function, preserved strength and sensation, intact coordination, and normal gait.

Initial laboratory evaluation revealed normocytic normochromic anemia (hemoglobin 9.8 g/dL at admission), consistent with the patient's underlying beta-thalassemia minor. Complete blood count demonstrated mild leukocytosis and reactive thrombocytosis with a platelet count of 499 × 10³/μL. The most striking abnormality was a severe cholestatic pattern of liver injury with alkaline phosphatase of 1252 U/L (normal range: 40-129 U/L), gamma-glutamyl transferase of 1426 U/L (normal range: 5-61 U/L), aspartate aminotransferase of 134 U/L, and alanine aminotransferase of 60 U/L. Total bilirubin increased progressively from 2.86 mg/dL to 5.35 mg/dL, predominantly the direct fraction (3.82 mg/dL), consistent with conjugated hyperbilirubinemia. Lactate dehydrogenase remained within normal limits. The patient demonstrated coagulopathy with an international normalized ratio (INR) of 1.7, which only partially corrected following vitamin K administration, suggesting synthetic liver dysfunction. Serum albumin was preserved at 4.5 g/dL. Mild hypercalcemia was noted with total calcium of 10.2 mg/dL (Table 1). Comprehensive infectious disease serologies were negative, including hepatitis B surface antigen, hepatitis C antibody, and human immunodeficiency virus antibody.

**Table 1:** Laboratory parameters at admission and post-treatment response *Beta-thalassemia minor baseline GGT, gamma-glutamyl transferase; ALT, alanine aminotransferase; AST, aspartate aminotransferase; INR, international normalized ratio; IgG, immunoglobulin G; M-protein, monoclonal protein; NT-proBNP, N-terminal pro-brain natriuretic peptide; hs-TnT, high-sensitivity cardiac troponin T; κ, kappa; λ, lambda

Laboratory parameters	At admission	Post-treatment	Reference range
Hemoglobin (g/dL)	9.8*	-	12.0-16.0
Platelets (×10³/μL)	499	-	150-400
Total bilirubin (mg/dL)	2.86 → 5.35	Normalized	0.2-1.2
Direct bilirubin (mg/dL)	3.82	-	0.0-0.3
ALT (U/L)	60	Normalized	4-34
AST (U/L)	134	Normalized	4-27
Alkaline phosphatase (U/L)	1252	412	40-129
GGT (U/L)	1426	687	5-61
Albumin (g/dL)	4.5	-	3.5-5.0
INR	1.7 → 1.75	<1.3	0.8-1.2
Creatinine (mg/dL)	Normal	Normal	0.6-1.2
Calcium (mg/dL)	10.2	Normalized	8.5-10.2
IgG (mg/dL)	1800	788	680-1450
M-protein (g/L)	1.2	0.3	-
Free κ light chain (mg/dL)	2.88	1.87	0.67-2.24
Free λ light chain (mg/dL)	16.1	9.25	0.83-2.70
κ/λ ratio	0.18	0.20	0.31-1.56
NT-proBNP (ng/L)	1142	-	<125
hs-TnT (ng/L)	26	-	<14
Beta-2 microglobulin (mg/L)	3.3	-	<2.4

Autoimmune hepatitis panel was negative, and alpha-1 antitrypsin deficiency was excluded (alpha-1 antitrypsin level 279.4 mg/dL). Renal function remained remarkably preserved with normal serum creatinine and estimated glomerular filtration rate. Urinalysis demonstrated no significant proteinuria, with an albumin-to-creatinine ratio of 21.18 mg/g and 24-hour urine protein quantification showing only 26.56 mg.

**Figure 1:**
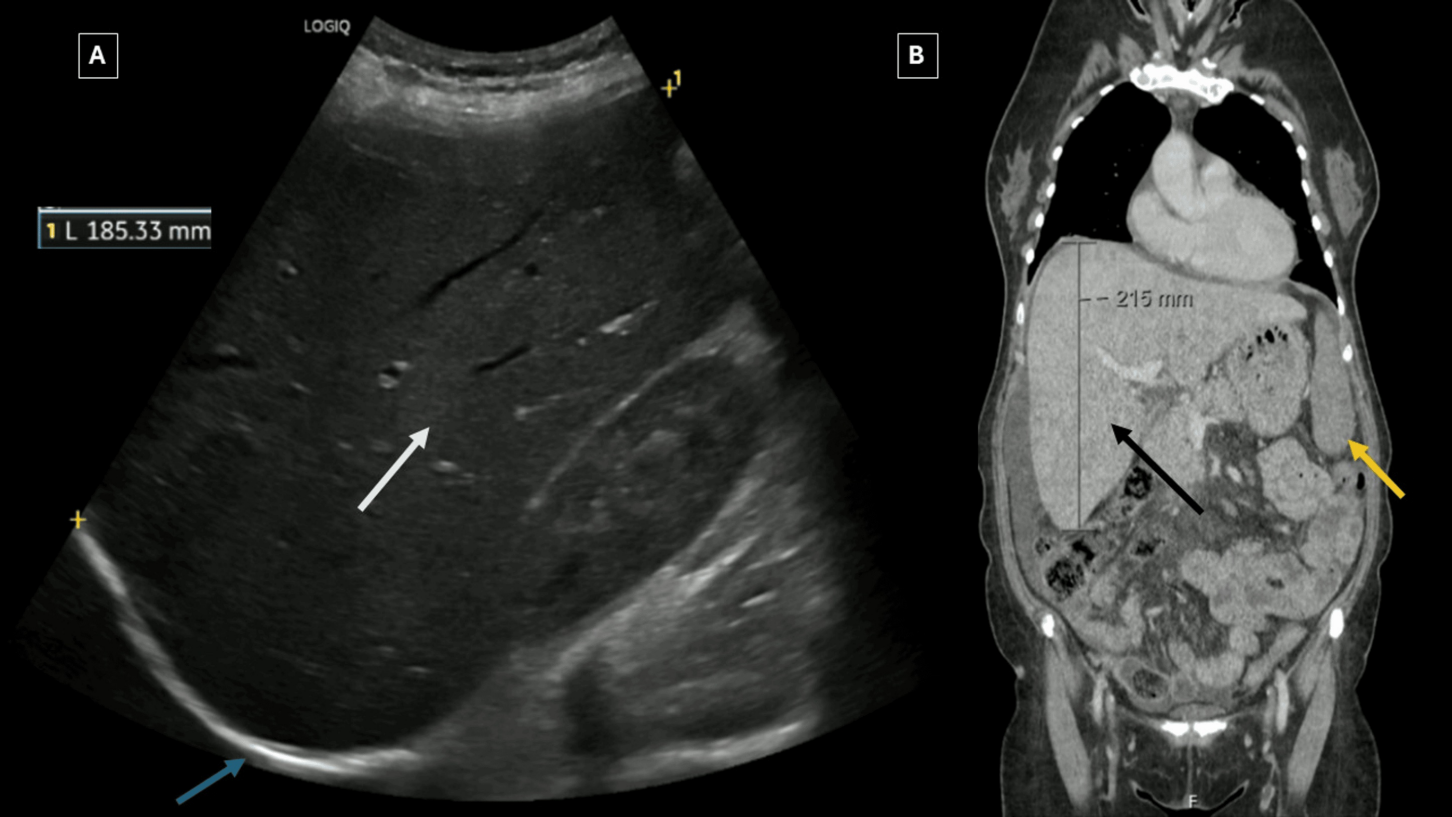
(A) Abdominal ultrasound showed hepatomegaly with homogeneous echotexture (white arrow) and rounded hepatic contours (blue arrow), raising concern for infiltrative hepatopathy. (B) Contrast-enhanced CT confirmed an enlarged liver with heterogeneous parenchymal enhancement (black arrow) and mild splenomegaly (yellow arrow).

During the six-month outpatient evaluation in the internal medicine clinic, progressive imaging studies had been obtained to investigate the patient's symptoms. Abdominal ultrasound demonstrated hepatomegaly with the right hepatic lobe measuring 18.5 cm in the mid-clavicular line (normal: less than 13 cm), globular hepatic morphology with rounded contours, and homogeneous echotexture with preserved echogenicity, raising concern for infiltrative hepatopathy (Figure 1). No focal hepatic lesions, bile duct dilatation, or ascites were identified at that time. 

CT of the abdomen and pelvis with intravenous contrast performed approximately one month after the initial ultrasound subsequently demonstrated chronic liver disease with hepatomegaly and heterogeneous hepatic parenchymal enhancement, suggesting "congestive hepatopathy." No hepatic masses or nodules were identified. The biliary tree showed mild prominence of the common bile duct, likely post-cholecystectomy change, without intrahepatic biliary dilatation. Mildly prominent pericardiophrenic and cardiomediastinal lymph nodes were noted. Small volume ascites was present (Figure 1).

**Figure 2:**
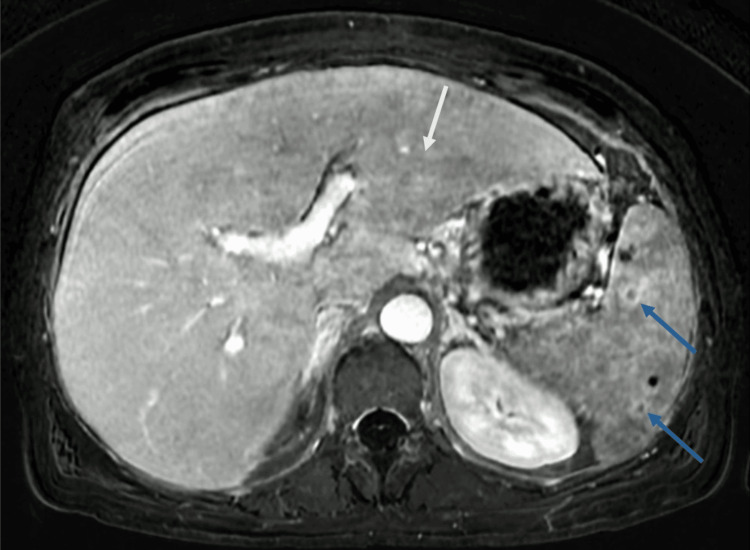
Magnetic resonance cholangiopancreatography demonstrated massive hepatomegaly with a globular morphology and diffuse heterogeneous contrast enhancement (white arrow), suggestive of confluent fibrosis. Mild splenomegaly with siderotic nodules (blue arrows) was also observed.

Magnetic resonance cholangiopancreatography was then performed to further characterize the hepatic findings and confirmed hepatomegaly with the right lobe measuring approximately 21 cm, globular morphology, and diffusely heterogeneous contrast enhancement with linear areas of peripheral enhancement showing progressive contrast uptake, potentially indicating confluent fibrosis (Figure 2). No focal hepatic lesions or biliary obstruction were identified. Mild splenomegaly was noted with a splenic diameter of 13.2 cm. Two accessory spleens measuring 18 mm and 19 mm were present adjacent to the splenic hilum. Multiple millimetric T1 hyperintense nodules within the spleen were consistent with siderotic nodules. Small volume ascites was again noted.

Following hospital admission, comprehensive serum protein studies were performed to investigate the unexplained hepatic dysfunction. Serum protein electrophoresis revealed multiple peaks in the alpha and gamma regions with an elevated erythrocyte sedimentation rate of 86 mm/hour. Quantitative immunoglobulin measurements demonstrated elevated IgG of 1800 mg/dL (normal range: 680-1450 mg/dL), with immunoglobulin A (IgA) of 145 mg/dL and immunoglobulin M (IgM) of 57 mg/dL within normal limits. Serum immunofixation electrophoresis identified a monoclonal IgG lambda protein with M-protein quantification of 1.2 g/L. Beta-2 microglobulin was elevated at 3.3 mg/L (normal range: less than 2.4 mg/L). Serum free light chain analysis revealed a markedly abnormal kappa/lambda ratio of 0.18 (normal range: 0.31-1.56), with kappa light chains of 2.88 mg/dL and lambda light chains of 16.10 mg/dL, confirming lambda light chain predominance (Table 1). Urine immunofixation electrophoresis demonstrated free lambda light chains with 24-hour urinary lambda excretion of 12 mg/day. Urine-free light chains were quantified at 7.6 mg/L.

**Figure 3:**
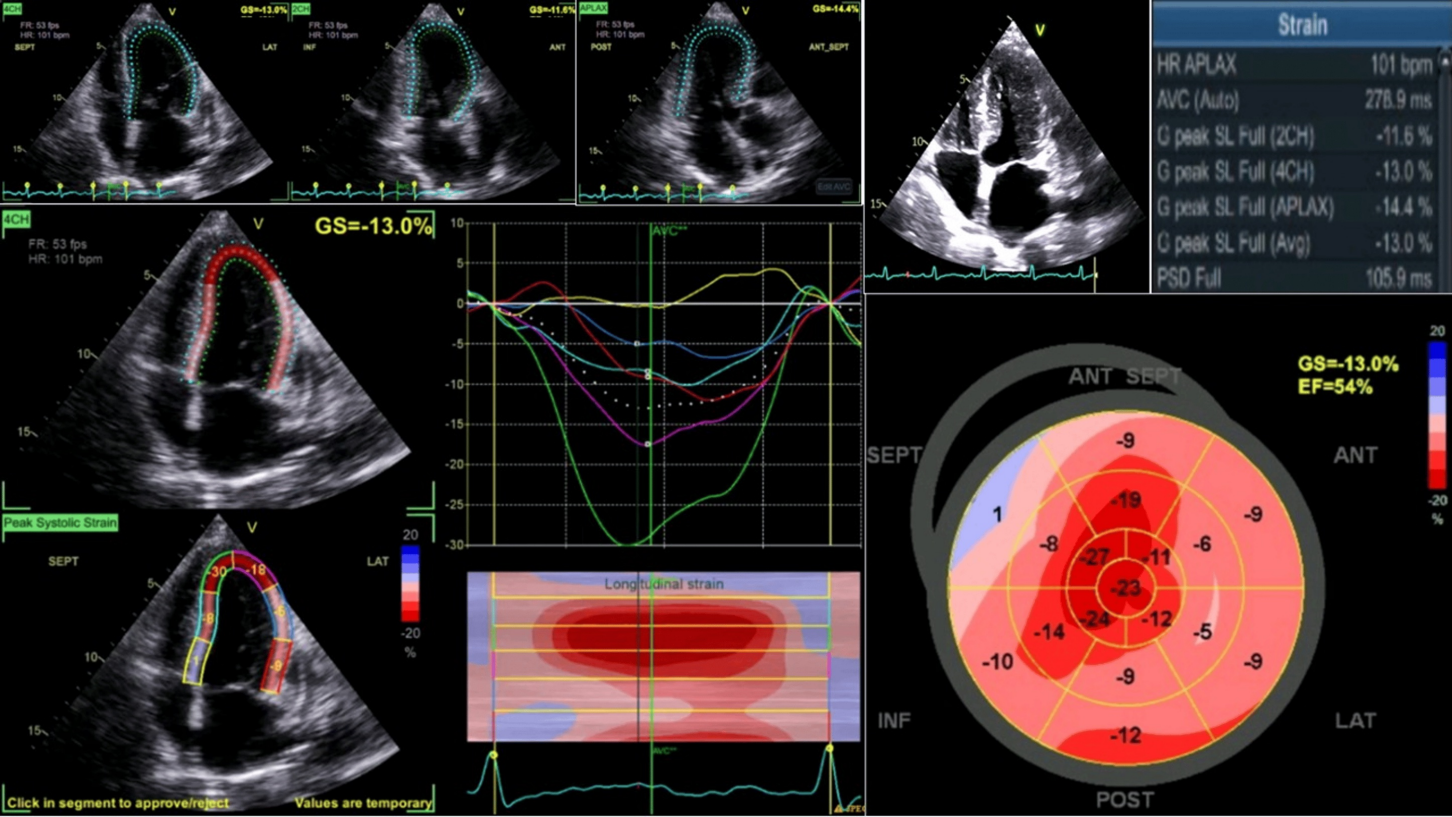
Transthoracic echocardiography revealed concentric left ventricular hypertrophy with preserved systolic function and a characteristic “apical sparing” pattern of global longitudinal strain, consistent with cardiac amyloidosis. HR, heart rate; PSD, peak strain dispersion; GS, global strain; EF, ejection fraction; ANT, anterior segment; INF, inferior segment; LAT, lateral segment; POST, posterior segment; SEPT, septal segment; G peak SL, global peak strain longitudinal; AVC, aortic valve closure; HR APLAX, heart rate at apical long-axis view; LVP, left ventricular pressure

Cardiac involvement was suggested by elevated N-terminal pro-brain natriuretic peptide (NT-proBNP) of 1142 ng/L (normal range: less than 125 ng/L) and high-sensitivity cardiac troponin T (hs-TnT) of 26 ng/L (normal range: less than 14 ng/L). Transthoracic echocardiography was performed and revealed findings highly suggestive of infiltrative cardiomyopathy. Left ventricular dimensions were normal with mild left atrial dilatation (left atrial volume index: 37 mL/m²) and concentric left ventricular moderate hypertrophy. All cardiac valves demonstrated normal morphology and function; left ventricular systolic function was globally preserved with an ejection fraction of 54% calculated by Simpson's biplane method, and no regional wall motion abnormalities were observed. However, global longitudinal strain was reduced at -13% (normal: greater than -18%) with a characteristic "apical sparing" pattern (Figure 3), in which basal and mid-ventricular segments showed reduced strain while apical segments were relatively preserved: a pattern highly specific for cardiac amyloidosis with reported sensitivity of 93% and specificity of 82% [10,11]. The apical sparing pattern is considered a hallmark finding of cardiac amyloidosis and was sufficient, combined with elevated biomarkers and consistent imaging, to establish stage II cardiac amyloidosis without requiring further cardiac magnetic resonance imaging. Minimal pericardial effusion was also present.

CT of the thorax, abdomen, and pelvis with bone windows was performed to evaluate for skeletal lesions and revealed no lytic or sclerotic osseous lesions. Diffuse trabecular heterogeneity suggested generalized osteopenia. Cardiomegaly with biventricular prominence was noted, and dependent ground-glass opacities suggested pulmonary edema or congestion.

**Figure 4:**
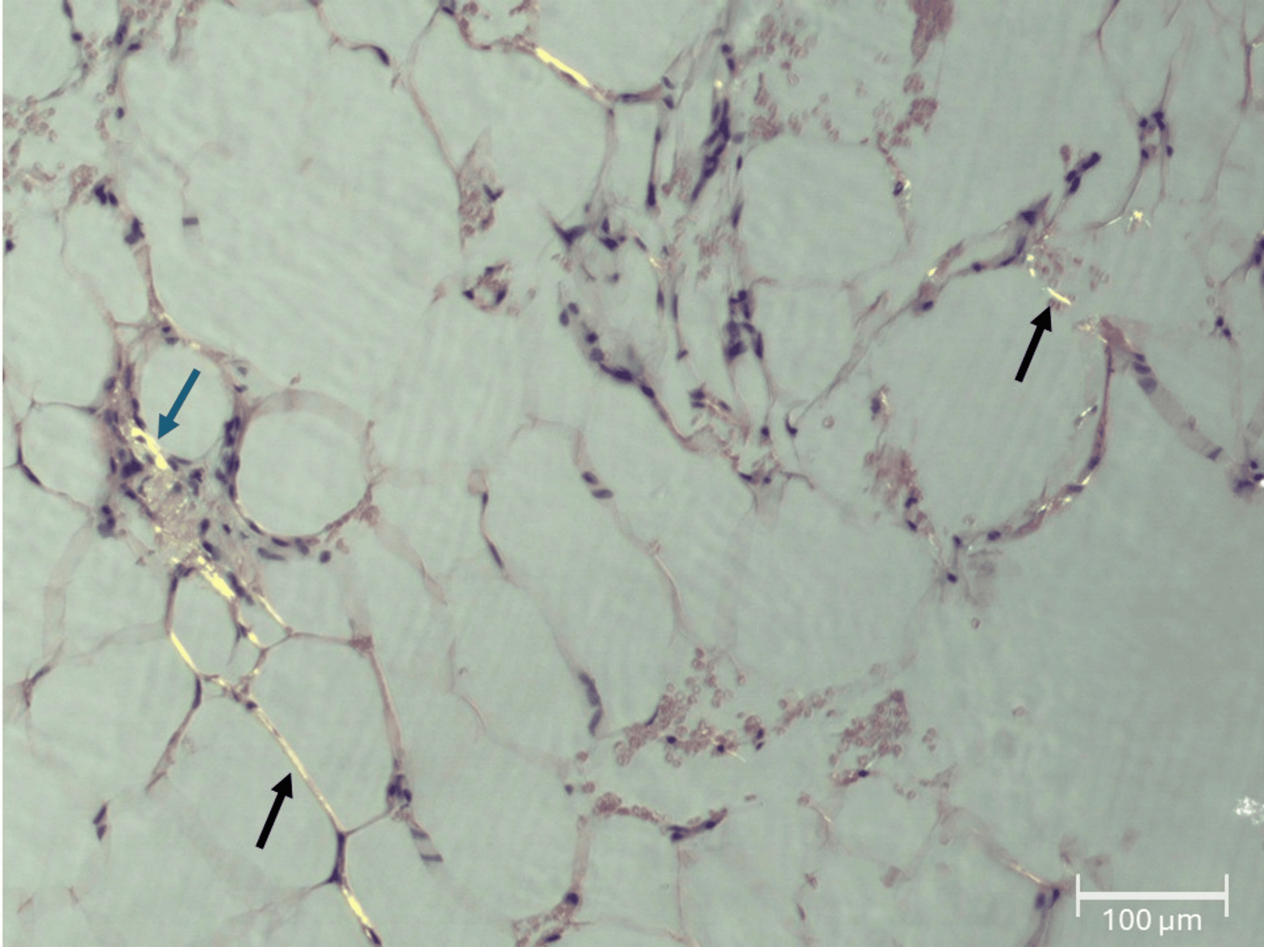
Subcutaneous abdominal fat pad aspiration biopsy showed adipose tissue containing blood vessels with mild wall thickening due to amyloid deposition (blue arrow). Congo red staining demonstrated characteristic apple-green birefringence (all arrows) under polarized light microscopy, confirming the presence of amyloid. Magnification: 10x

Given the constellation of clinical findings, there was high suspicion for systemic amyloidosis with hepatic involvement. To establish a definitive diagnosis and guide appropriate treatment, tissue confirmation of amyloidosis was deemed essential. Subcutaneous abdominal fat pad aspiration biopsy was performed on day 3 of hospitalization. Histopathological examination revealed adipose tissue containing blood vessels with mild wall thickening due to amyloid deposition. Congo red staining demonstrated characteristic apple-green birefringence under polarized light microscopy, confirming the presence of amyloid (Figure 4). Immunofluorescence studies showed deposition of lambda light chains with an absence of kappa light chains and heavy chains (IgG, IgA, and IgM), establishing the diagnosis of lambda light chain (AL) amyloidosis.

**Figure 5:**
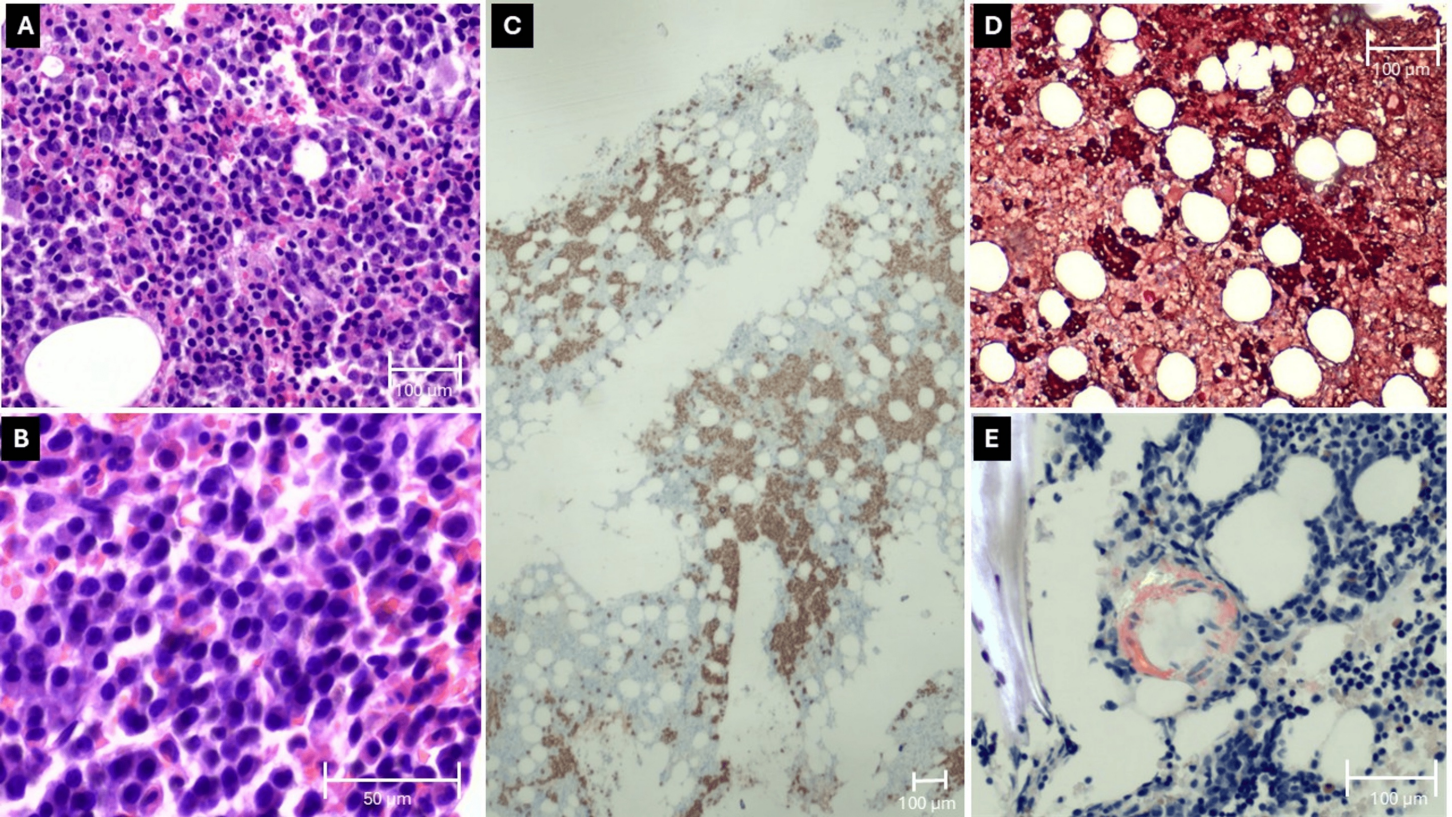
(A, B) Bone marrow aspirate showed plasma cells comprising approximately 30% of marrow cellularity with abnormal morphology and a characteristic “wrapping” appearance, consistent with neoplastic transformation (hematoxylin and eosin staining). (C) CD138 expression confirmed plasma cell lineage. (D) Immunohistochemistry revealed predominantly lambda light chain-restricted plasma cells (lambda-brown/kappa-red dual staining). (E) Congo red staining identified amyloid deposition within the microvasculature. Magnification: (A) 10x; (B) 40x; (C) 2x; (D) 10x; (E) 20x

Bone marrow aspiration and biopsy were obtained from the posterior iliac crest on day 7. Histopathological examination revealed hypercellular marrow (80% cellularity, 20% adipose tissue), with a substantial increase in plasma cells constituting approximately 30% of total marrow cellularity. The plasma cell population demonstrated significant cytomorphologic atypia with characteristic wrapping morphology and formed cohesive aggregates. Immunophenotypic characterization by immunohistochemistry and flow cytometry demonstrated CD138 expression, CD38 (weak intensity), and CD56 expression (weak intensity), with predominant lambda light chain restriction. Congo red staining identified amyloid substance deposited within the microvasculature (Figure 5). The constellation of findings, including a clonal plasma cell proliferation with lambda light chain restriction, characteristic wrapping morphology, bone marrow involvement exceeding 10%, and histological evidence of amyloid deposition, fulfilled diagnostic criteria for multiple myeloma with concurrent AL amyloidosis as defined by the International Myeloma Working Group [12].

An initial liver biopsy was considered and requested via the transjugular route to minimize bleeding risk, given the patient's coagulopathy. However, scheduling constraints prevented the performance of the procedure during the initial hospitalization. Subsequently, after confirmation of AL amyloidosis via abdominal fat pad biopsy and given the clinical constellation of hepatomegaly, cholestasis, and coagulopathy in the setting of proven systemic amyloidosis, the liver biopsy was deemed unnecessary and was cancelled. This decision was justified by several factors: the established diagnosis of systemic AL amyloidosis through fat pad biopsy with immunofluorescence showing lambda light chain restriction; characteristic imaging features of hepatic infiltration with progressive heterogeneous enhancement; the presence of marked cholestasis and coagulopathy indicating advanced hepatic involvement; and the substantial bleeding risk in this patient due to both INR elevation (1.7) and the inherent vascular fragility associated with amyloid-infiltrated liver tissue. This clinical approach is supported by published literature demonstrating that liver biopsy can be avoided when the diagnosis of systemic amyloidosis is established elsewhere, and clinical and radiological features are consistent with hepatic involvement [9]. Furthermore, liver biopsy in this setting carries increased bleeding risk due to both coagulopathy and the increased fragility of amyloid-infiltrated blood vessels. While a transjugular approach would reduce bleeding risk compared to percutaneous biopsy, the diagnostic information gained would be redundant given the already established diagnosis of systemic AL amyloidosis.

Based on the revised prognostic staging system for light chain amyloidosis, the patient was classified as cardiac stage II, with NT-proBNP of 1142 ng/L (stage I: NT-proBNP less than 1800 ng/L and troponin less than 25 ng/L; stage II: either NT-proBNP greater than or equal to 1800 ng/L or troponin greater than or equal to 25 ng/L; stage III: both criteria met) [13]. The presence of elevated cardiac biomarkers with echocardiographic evidence of infiltrative cardiomyopathy confirmed cardiac involvement. For multiple myeloma, the patient was classified as International Staging System (ISS) stage I based on beta-2 microglobulin of 3.3 mg/L (less than 3.5 mg/L) and preserved albumin of 4.5 g/dL (ISS I: beta-2 microglobulin less than 3.5 mg/L and albumin greater than or equal to 3.5 g/dL; ISS II: neither stage I nor stage III; ISS III: beta-2 microglobulin greater than or equal to 5.5 mg/L) [14].

Following diagnostic confirmation, treatment was initiated with daratumumab in combination with cyclophosphamide, bortezomib, and dexamethasone (Dara-VCD). The treatment protocol consisted of 28-day cycles with daratumumab 1800 mg subcutaneously, bortezomib 0.7 mg/m² subcutaneously (dose-reduced due to neuropathy risk and performance status), cyclophosphamide 500 mg orally, and dexamethasone 20 mg orally on days 1, 8, 15, and 22, with dexamethasone 4 mg on days 2, 9, 16, and 23. Prophylactic antimicrobial therapy was initiated with trimethoprim-sulfamethoxazole for* Pneumocystis jirovecii *prophylaxis and aciclovir for herpes simplex virus and varicella-zoster virus prophylaxis.

The first dose of Dara-VCD was administered on hospital day 18. Shortly after, the patient developed febrile syndrome with methicillin-sensitive *Staphylococcus aureus* bacteremia, leading to treatment suspension. Despite clearance of bacteremia after starting flucloxacillin, fever and inflammatory markers persisted. Exhaustive infectious diseases workup ruled out localized infection; peripheral venous catheter thrombophlebitis was presumed source and was resolved after catheter removal. Subsequently, urinary tract infection with* Klebsiella pneumoniae *bacteremia was documented and treated with 14 days of piperacillin/tazobactam, leading to improvement. The development of these infections reflects the profound immunosuppression induced by daratumumab-based therapy, which depletes CD38-expressing B cells and plasma cells, substantially increasing infection risk, particularly for encapsulated organisms and gram-negative bacteria. The use of antimicrobial prophylaxis (trimethoprim-sulfamethoxazole and aciclovir) was appropriate for this high-risk population, though breakthrough infections occurred. Following infection clearance, careful reassessment was undertaken before resuming chemotherapy to balance infection control against continued disease control and prevent amyloid-mediated organ progression.

Chemotherapy was reinitiated on hospital day 58, and the patient was discharged two days later. Following discharge, after four cycles of Dara-VCD chemotherapy over approximately five months, serial laboratory studies showed a significant hematologic response. M-protein decreased from 1.2 g/L to 0.3 g/L, representing a 75% reduction, which corresponds to a very good partial response (VGPR) by International Myeloma Working Group criteria [15]. Serum free light chain analysis showed a decrease in lambda light chain from 16.1 mg/dL to 9.25 mg/dL with an improved kappa/lambda ratio from 0.18 to 0.20. Organ response was evidenced by normalization of liver function tests: alkaline phosphatase decreased from 1252 U/L to 412 U/L, gamma-glutamyl transferase from 1426 U/L to 687 U/L, and total bilirubin normalized. Hepatic function improvement was accompanied by clinical improvement with resolution of jaundice and regaining of functional independence. The patient tolerated oral intake with only mild chemotherapy-related nausea and maintained improved functional status at follow-up. Additional long-term follow-up data regarding cardiac biomarker response and imaging reassessment are pending completion of the planned treatment course.

## Discussion

This case exemplifies a clinically significant presentation combining multiple uncommon features: IgG lambda-type multiple myeloma, systemic AL amyloidosis, and dominant hepatic involvement as the initial clinical manifestation. While each of these elements individually represents a relatively uncommon scenario, their confluence in a single patient makes this case instructive for clinical practice. Hepatic amyloidosis, while frequently detected histologically at autopsy, rarely dominates the clinical presentation [16]. In a recent cohort study of 88 patients with systemic AL amyloidosis and hepatic involvement, hepatic-predominant disease with significant clinical manifestations represented a recognizable but minority subset of cases [9]. The typical immunoglobulin profile differs substantially from what was observed in this case. In multiple myeloma overall, IgG kappa is the most common subtype, occurring approximately twice as frequently as IgG lambda [8]. While lambda light chains are more common than kappa in AL amyloidosis generally (occurring in 70-75% of cases), the specific combination of IgG lambda multiple myeloma with hepatic-predominant AL amyloidosis and preserved renal function remains clinically noteworthy [5].

The diagnostic odyssey in this case highlights several important clinical lessons. The patient's six-month prodrome of constitutional symptoms prompted extensive outpatient evaluation before the correct diagnosis was established. This diagnostic delay is characteristic of hepatic amyloidosis, which often masquerades as more common hepatobiliary conditions. In one large series, amyloidosis was suspected in only 26% of cases prior to liver biopsy, and up to 40% of patients consulted five or more physicians before diagnosis [6]. The cholestatic pattern of liver injury with markedly elevated alkaline phosphatase and gamma-glutamyl transferase out of proportion to transaminase elevation is characteristic of hepatic amyloidosis. The progressive conjugated hyperbilirubinemia and coagulopathy, indicating synthetic dysfunction, suggested advanced hepatic involvement.

The preserved albumin level in this case (4.5 g/dL) was somewhat atypical, as albumin is frequently reduced in advanced hepatic amyloidosis [7]. This discordance between markers of synthetic function can be explained by several mechanisms: albumin has a long half-life of approximately 20 days, so acute hepatic dysfunction may not yet be reflected in albumin levels; infiltrative hepatopathies like amyloidosis may impair specific hepatic functions (cholestasis, coagulation factor synthesis) before affecting overall hepatic protein synthesis; and the patient's relatively early clinical presentation, before terminal hepatic decompensation, may explain preservation of some synthetic functions despite advanced infiltration. The heterogeneous hepatomegaly noted on imaging raised initial concern for malignancy, cirrhosis, or infiltrative disease. The initial description of "congestive hepatopathy" on contrast-enhanced CT likely reflected the imaging appearance of infiltrative hepatic disease with heterogeneous enhancement, rather than indicating true venous congestion from right-sided heart failure. Although the patient had stage II cardiac amyloidosis, the cardiac dysfunction at presentation was not severe enough to cause significant right-sided failure and hepatic congestion; imaging findings were consistent with primary amyloid hepatic infiltration.

The diagnosis of AL amyloidosis requires demonstration of amyloid deposits in affected tissues combined with evidence of a clonal plasma cell disorder. In this case, the abdominal fat pad biopsy provided rapid, minimally invasive confirmation of amyloid deposition. Abdominal fat aspiration has a sensitivity of 70-85% for detecting systemic amyloidosis and is typically the first-line biopsy site due to its accessibility and low complication rate [16]. Congo red staining demonstrating apple-green birefringence under polarized light confirmed the presence of amyloid, while immunofluorescence studies establishing lambda light chain positivity confirmed the AL subtype. Although liver biopsy would have definitively confirmed hepatic amyloid involvement, the combination of proven systemic AL amyloidosis, marked hepatomegaly, severe cholestasis, coagulopathy, and characteristic imaging findings provided sufficient evidence for hepatic involvement without requiring direct liver tissue sampling.

The bone marrow biopsy demonstrating 30% plasma cell infiltration established the diagnosis of multiple myeloma according to International Myeloma Working Group criteria [12]. The pattern of organ involvement in this case is noteworthy for several reasons. Cardiac involvement, evidenced by elevated NT-proBNP, elevated troponin, and characteristic echocardiographic findings of reduced global longitudinal strain with apical sparing, confirmed cardiac amyloidosis. The apical sparing pattern on strain imaging has emerged as a highly sensitive and specific marker for cardiac amyloidosis with reported sensitivity of 93% and specificity of 82% [10,11]. The absence of renal involvement is particularly remarkable and represents a notable deviation from the typical presentation of AL amyloidosis. Renal involvement typically occurs in approximately 70% of AL amyloidosis cases [1]. Similarly, the absence of lytic bone lesions on skeletal imaging distinguishes this case from typical presentations of multiple myeloma, in which skeletal involvement occurs in up to 80% of patients [5].

The treatment strategy employed in this case reflects contemporary evidence-based approaches to systemic AL amyloidosis with associated multiple myeloma. Daratumumab, a human monoclonal antibody targeting CD38, has revolutionized the treatment of both multiple myeloma and AL amyloidosis [16]. The ANDROMEDA trial demonstrated superior outcomes with daratumumab-based regimens compared to historical cyclophosphamide-bortezomib-dexamethasone alone in newly diagnosed AL amyloidosis [17,18]. Bortezomib is particularly effective in plasma cell dyscrasias due to its ability to induce apoptosis in malignant plasma cells [19]. The dose reduction of bortezomib in this case was appropriate given the patient's age, performance status, and risk of peripheral neuropathy.

The hematologic response achieved with M-protein reduction from 1.2 g/L to 0.3 g/L (75% reduction) corresponds to VGPR as per standardized International Myeloma Working Group criteria [15]. Organ response was demonstrated by normalization of liver function markers (alkaline phosphatase from 1252 to 412 U/L, gamma-glutamyl transferase from 1426 to 687 U/L, and total bilirubin normalization) and clinical recovery of hepatic function. This degree of response is associated with improved organ function recovery and prolonged survival in AL amyloidosis [15,17]. The infectious complications encountered during treatment, methicillin-sensitive *Staphylococcus aureus* bacteremia and *Klebsiella pneumoniae* urinary tract infection with bacteremia, underscore the profound immunosuppression induced by this chemotherapy regimen. Daratumumab depletes CD38-expressing immune cells, including normal B cells and plasma cells, predisposing to both bacterial and viral infections [18]. The development of bacteremia necessitated treatment interruption and extended antibiotic therapy. These complications are well-documented in patients receiving daratumumab-based regimens and represent a known risk-benefit consideration when initiating therapy [18].

The prognosis for patients with AL amyloidosis and multiple myeloma has improved substantially with modern therapies, but remains guarded. Historical data from the pre-daratumumab era demonstrated a median survival of only eight to nine months in patients with hepatic involvement [16]. However, contemporary series using novel agent-based regimens report significantly improved outcomes. Cardiac involvement remains the most important prognostic determinant [20]. This patient's stage II cardiac disease places her in an intermediate risk category. The absence of renal involvement is prognostically favorable. The achievement of rapid and deep hematologic response is also associated with improved organ response and survival [16,18].

This case has inherent limitations that should be acknowledged. First, the diagnosis of hepatic AL amyloidosis was not confirmed by direct liver tissue examination, though the combination of established systemic amyloidosis, characteristic imaging, and clinical features of hepatic dysfunction provided strong supporting evidence. Second, follow-up duration has been modest (approximately five months post-initiation of therapy), limiting statements regarding long-term prognosis and durability of response. Third, this is a single-patient case, and observations regarding regimen effectiveness are illustrative for this individual and should not be generalized beyond existing randomized controlled trial data. Long-term monitoring of cardiac biomarkers and imaging reassessment are planned as part of ongoing clinical care.

## Conclusions

We report a clinically significant case of IgG lambda multiple myeloma complicated by systemic AL amyloidosis presenting with dominant hepatic involvement manifesting as severe cholestatic jaundice, hepatomegaly, and coagulopathy. The patient's diagnostic evaluation revealed 30% bone marrow plasma cell infiltration, lambda light chain amyloid deposition confirmed by abdominal fat pad biopsy, and evidence of cardiac involvement with preserved renal function and absence of skeletal lesions. Treatment with Dara-VCD resulted in VGPR with a 75% reduction in M-protein and significant improvement in hepatic function despite infectious complications requiring treatment interruption. This case emphasizes the importance of maintaining clinical suspicion for AL amyloidosis in patients with unexplained multi-organ dysfunction, highlights the value of systematic diagnostic evaluation, including minimally invasive biopsy techniques and advanced cardiac imaging, and demonstrates the effectiveness of modern anti-plasma cell therapies in achieving disease control even in rare and complex presentations. Early recognition and prompt initiation of appropriate therapy are essential for optimizing outcomes in this challenging condition.
